# Myeloma Bone Disease: A Comprehensive Review

**DOI:** 10.3390/ijms22126208

**Published:** 2021-06-08

**Authors:** Shiva Kumar Reddy Mukkamalla, Dhatri Malipeddi

**Affiliations:** 1 Hematology/Medical Oncology, Presbyterian Healthcare Services, Rio Rancho, NM 87124, USA; 2 Internal Medicine, Canton Medical Education Foundation/NEOMED, Canton, OH 44710, USA; dhatrim@gmail.com

**Keywords:** multiple myeloma, bone disease, therapies

## Abstract

Multiple myeloma (MM) is a neoplastic clonal proliferation of plasma cells in the bone marrow microenvironment, characterized by overproduction of heavy- and light-chain monoclonal proteins (M-protein). These proteins are mainly found in the serum and/or urine. Reduction in normal gammaglobulins (immunoparesis) leads to an increased risk of infection. The primary site of origin is the bone marrow for nearly all patients affected by MM with disseminated marrow involvement in most cases. MM is known to involve bones and result in myeloma bone disease. Osteolytic lesions are seen in 80% of patients with MM which are complicated frequently by skeletal-related events (SRE) such as hypercalcemia, bone pain, pathological fractures, vertebral collapse, and spinal cord compression. These deteriorate the patient’s quality of life and affect the overall survival of the patient. The underlying pathogenesis of myeloma bone disease involves uncoupling of the bone remodeling processes. Interaction of myeloma cells with the bone marrow microenvironment promotes the release of many biochemical markers including osteoclast activating factors and osteoblast inhibitory factors. Elevated levels of osteoclast activating factors such as RANK/RANKL/OPG, MIP-1-α., TNF-α, IL-3, IL-6, and IL-11 increase bone resorption by osteoclast stimulation, differentiation, and maturation, whereas osteoblast inhibitory factors such as the Wnt/DKK1 pathway, secreted frizzle related protein–2, and runt-related transcription factor 2 inhibit osteoblast differentiation and formation leading to decreased bone formation. These biochemical factors also help in development and utilization of appropriate anti-myeloma treatments in myeloma patients. This review article summarizes the pathophysiology and the recent developments of abnormal bone remodeling in MM, while reviewing various approved and potential treatments for myeloma bone disease.

## 1. Introduction

Multiple myeloma (MM) is a neoplastic clonal proliferation of plasma cells in the bone marrow microenvironment, characterized by overproduction of heavy- and light-chain monoclonal proteins (M-protein) [1,2]. These proteins are mainly found in the serum and/or urine. Reduction in normal gammaglobulins (immunoparesis) leads to an increased risk of infection [3]. MM is a relatively uncommon cancer, but it is the second most common hematologic malignancy. MM comprises approximately 2% of all cancers in the US and about 15% of lymphohematopoietic cancers (LHC). In the United States, the lifetime risk of getting MM is 1 in 132 (0.76%). The American Cancer Society estimates that about 34,920 new cases of MM will be diagnosed in the United States for 2021 (19,320 in men and 15,600 in women). About 12,410 deaths are expected to occur (6840 in men and 5570 in women) [4]. This malignancy is seen more commonly in men over the age of 40 years, especially in men who belong to the African American ethnicity. Globally, approximately 86,000 new cases are seen annually, which accounts for 0.8% of all new cancer cases, and there are 63,000 deaths from this disease annually, accounting for 0.9% of all cancer deaths. Globally predominant areas of MM with the highest incidence include the industrialized regions of Australia/New Zealand, Europe, North America, and Asia [5].

We present a systematic review of clinical trials and various preclinical studies, identified through a comprehensive search in using PubMed. Our main goal is to discuss in depth the pathogenesis of bone disease in MM and investigate and critically examine the effects of various treatments that have recently been approved by the US Food and Drug Administration over the last two decades in the management of myeloma bone disease or therapies that are being investigated.

## 2. Pathogenesis

Patients with MM usually present with hypercalcemia, anemia, renal damage, increased risk for infections, and pathological fracture secondary to osteolytic bone destruction [1,2]. The osteolytic bone disease results from the disruption between the osteoclast, osteoblasts, bone marrow stromal cells, and osteocytes [6,7,8]. Gradual worsening of myeloma bone disease (MBD) presents with excruciating pain, pathological fractures, and symptomatic hypercalcemia [1]. MBD resulting in pathological fractures ultimately leads to poor quality of life secondary to the pain and hypercalcemia consequences. The etiology for the excessive bone mass loss seen in MM is multifactorial. Approximately 80% of patients with MM initially present with abnormal bone structure at the time of diagnosis [1,2]. In a 2003 study by Kyle et al., 67% of patients had osteolytic bone disease and 20% had osteoporosis with pathological fractures at diagnosis. Approximately 60% of MM patients develop a fracture during their disease course [9]. The severity of the bone disease is proportionate with the tumor burden. There is an inverse relationship between the number of osteolytic bone lesions and prognosis [10].

### 2.1. Normal Bone Remodeling

Bone remodeling occurs on the bone surface where the osteoclasts and osteoblasts are covered by the BRC canopy. The BRC is the space between the bone surfaces which is undergoing remodeling in the canopy of flattened cells. In adult bone, osteocytes comprise 90–95% of cells, whereas osteoclast and osteoblastic cells account for fewer than 10% of them [11]. Osteocytes function as the main regulators of bone homeostasis between osteoclast and osteoblasts [3]. Osteocytes secrete cytokines including sclerostin, DKK1/Wnt pathway inhibitor, RANKL, and OPG. These osteocytes contribute to the activation of bone remodeling process. They respond to mechanical stimulation and initiate bone resorption. Osteocyte death also results in the recruitment of osteoclasts.

**Figure 1:**
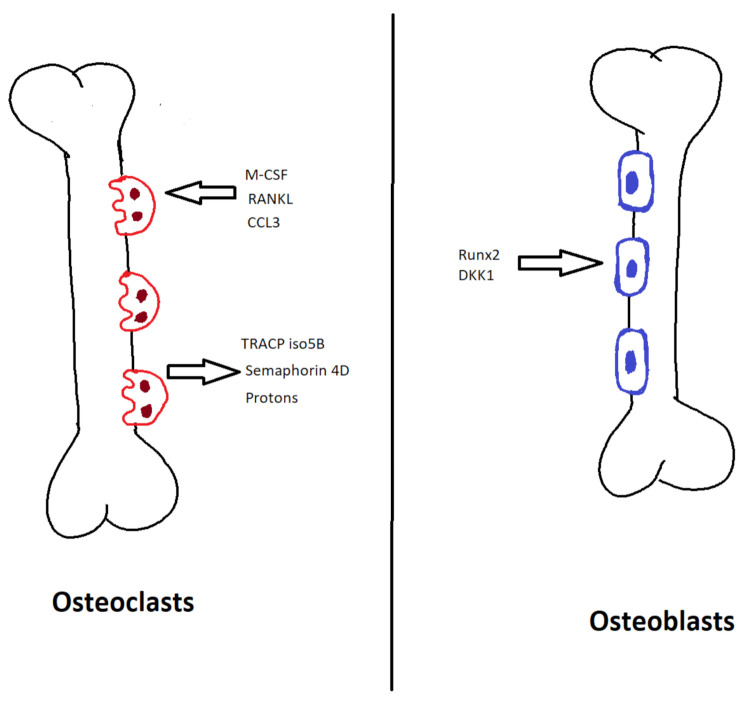
Normal bone remodeling. M-CSF, monocyte/macrophage colony stimulating factor; RANKL, receptor-activated nuclear factor-kappa B ligand; CCL3, C–C motif chemokine ligand 3; TRACP iso5B, tartrate-resistant acid phosphatase 5b; Runx2, runt-related transcription factor 2; DKK1, dickkopf WNT signaling pathway inhibitor 1.

Osteoclasts are multinucleated cells that originate from the monocyte-macrophage cell lineage and cause bone resorption. Monocyte/macrophage colony stimulating factor (M–CSF) and RANKL are responsible for the differentiation of the precursor cells into mature osteoclasts [12]. M-CSF causes osteoclastogenesis, whereas RANKL helps the differentiation and activation into mature osteoclast [13]. During osteoclast development, tartrate-resistant acid phosphatase 5b (TRACP isotype 5B) is formed, a biomarker specific to osteoclasts. During osteoclastogenesis, large multinucleated cell formation occurs, causing bone degradation by active secretion of protons into the resorption pits, which decreases the pH leading to decalcification of the bone matrix [14]. Furthermore, the collagen fibers are degraded by the proteolytic enzyme cathepsin K and various matrix metalloproteinases [15]. Osteoclasts also express semaphorin 4D (SEMA4D), which inhibits osteoblastic differentiation [16] (Figure 1).

Osteoblasts originate from the mesenchymal stem cell. They are responsible for the new bone formation following bone resorption by the osteoclast. Runt-related transcription factor 2 (Runx2) is responsible for the differentiation of mesenchymal stem cells into osteoblasts [17]. This is followed by the Wnt pathway, which increases β-catenin but helps the differentiation and maturation of osteoblasts. The Wnt pathway is mediated by a complex formation that inhibits the degradation of β-catenin. Wnt pathway is inhibited by Dickkopf 1(DKK1) leading to bone formation [18]. These mature osteoblasts are located along the newly resorbed bone and generate the bone matrix, mainly collagen type I, which is followed by calcification to complete the process of bone formation [19]. During bone formation, some osteoblasts are incorporated into the bone matrix and convert into osteocytes. Osteocytes cause osteoblast differentiation via sclerostin and DKK1 which inhibit the Wnt pathway by binding to LDL receptor-related proteins 5 and 6 (Wnt receptors) on the surface of osteoblasts [11] (Figure 1).

### 2.2. Abnormal Bone Remodeling in MM

Normal bone remodeling is attained by the homeostasis between several factors balancing bone formation versus bone degradation. In MM, the osteocyte–osteoclast–osteoblast axis is disrupted, leading to the formation of pathognomonic osteolytic lesions [3]. One of the early events in MM is increased bone degradation. Increases in the number and the activity of osteoclast along with inadequate compensation by osteoblasts in patients with MM have been found, resulting in osteolytic lesions when bone formation.

### 2.3. Bone Marrow Microenvironment, Myeloma Cells, T Lymphocytes and Bone Marrow Stromal Cells (BMSC)

The main pathophysiology behind the occurrence of MBD is increased bone degradation along with impaired bone formation. In monoclonal gammopathy of undetermined significance (MGUS) and early stages of MM, bone architecture is preserved. Occasionally, increased bone formation is seen. With the advanced staging of MM, impaired bone formation along with osteolytic lesions are observed.

The start of pathologic process involves the interaction between the bone marrow microenvironment and the myeloma cells. The bone remodeling compartment (BRC) canopy is a layer of flat cells belonging to osteoblastic lineage that separates the bone from the bone surface during bone remodeling, and it plays an important role in the remodeling process [20,21]. Destruction of the BRC canopy allows the myeloma cells to interact with the bone remodeling cells, causing a discrepancy in the bone homeostasis [22].

Interaction between myeloma cells, lymphocytes, and BMSC within the bone marrow microenvironment plays a role in the development of MBD [23]. Myeloma cells bind to BMSC via very late antigen 4 (VLA-4) and vascular cell addition molecule 1 (VCAM-1). This leads to the secretion of cytokines promoting osteoclast differentiation and activation [3]. The imbalanced ratio of RANKL and OPG (31) and chemokines such as MIP-1-α, IL-5, IL-3, IL-6, IL-7, SDF-1-α, and VEGF are responsible for increased osteoclastogenesis [7]. MM cells also stimulate CCL3 and IL-11 in osteocytes stimulating, osteoclast differentiation and activation [24].

Myeloma cells produce decoy receptor 3 (DCR 3) and are responsible for osteoclast differentiation and activation [25]. DCR3, which belongs to the TNF receptor support family, is overexpressed on myeloma cells and lymphocytes [26]. Myeloma cells also stimulate BMSC and osteoblasts to activate the RANKL/OPG system [27]. Myeloma cells interact with osteoclasts and can alter themselves into multinucleated cells with bone resorptive properties [28].

Myeloma cells adhere to BMSC, which regulates the production of RANKL, IL-6, B-cell activating factor (BAFF), and activin A [29,30]. BAFF belongs to the TNF superfamily, which plays a role in the development of B cells as well as promotes osteoclastogenesis and myeloma cell survival. Activin A belongs to the transforming growth factor (TGF) family and acts by activating osteoclast and inhibiting osteoblasts. High levels of activin A are noticed in an advanced stage of MM [31].

T-lymphocytes also play a role in regulating osteoclast/osteoblast activity, survival, and function [25]. Prabhala et al. demonstrated a subset of T helper cells called Th17-1 cells which secreted IL-17 that not only mediated myeloma bone disease but also increased myeloma cell survival [32,33]. Activated T cells and MM produce osteoclastogenic cytokines such as IL-3, RANKL, DCR 3, and TNF, enhancing osteolysis [26].

### 2.4. Increased Osteoclastogenesis

**Figure 2:**
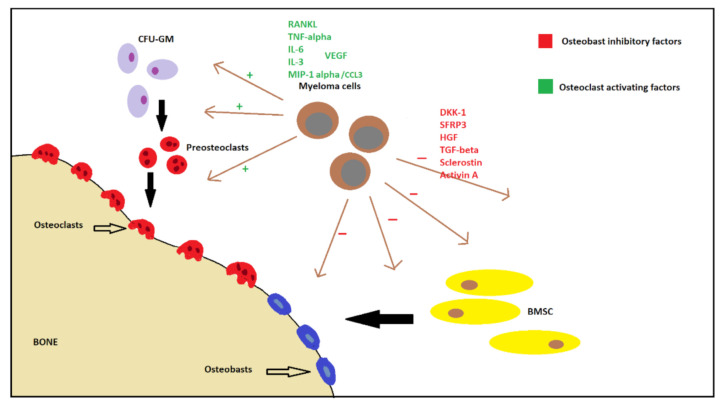
Mechanisms of myeloma-related bone disease. CFU-GM, colony forming unit—granulocyte/macrophage; RANKL, receptor-activated nuclear factor-kappa B ligand; TNF-α, tumor necrosis factor alpha; IL-6, interleukin-6; IL-3, interleukin-3; MIP-1-α (CCL3), macrophage inflammatory protein-1 alpha (C–C motif chemokine ligand 3); VEGF, vascular endothelial growth factor; DKK1, dickkopf WNT signaling pathway inhibitor 1; SFRP3, secreted frizzle related protein 3; HGF, hepatocyte growth factor; TGF-β, transforming growth factor beta; BMSC, bone marrow stromal cells.

Increases of osteoclast activity and bone resorption markers play a major role in myeloma bone disease [34,35]. Important biochemical markers involved in osteoclast activity and differentiation include RANKL/OPG [36,37,38,39] and decoy receptor 3(DcR3) [26,40,41]. Other chemokines include C–C motif chemokine ligand 3 (CCL3, also referred to as MIP-1-α), MIP-1-β [42,43,44], TNF-α [45,46], IL-3, IL-6, IL-11, stromal cell-derived factor 1 alpha (SDF-1-α), B-cell activating factor (BAFF), activin A, and vascular endothelial growth factor (VEGF) [3] (Figure 2).

### 2.5. RANKL/OPG System

In MM, disruption of the BRC canopy by the myeloma cells impairs the process of bone remodeling [22]. This is regulated by several factors but mainly by RANKL, RANK, and decoy receptor osteoprotegerin (OPG), which help maintain bone remodeling homeostasis. RANK is a transmembrane receptor that belongs to the TNF superfamily. It is produced by BMSC, osteoblasts, and activated T lymphocytes [47]. RANKL is a cytokine expressed as a membrane-bound protein by BMSCs of osteoblastic lineage and activated T lymphocytes. The RANK/RANKL/OPG system plays a major role in the development of myeloma bone disease. In normal individuals, the bone homeostasis is well-maintained by the RANKL/OPG system. However, in MM, the RANKL/OPG ratio is increased by an increase in RANKL and decrease in OPG, ultimately resulting in increased bone resorption [38]. The severity of the ratio is directly proportional to the overall survival/prognosis of the disease. An increase in the RANKL:OPG ratio can cause bone loss or increased resorption in various malignancies and non-malignant inflammatory disorders, such as rheumatoid arthritis [48,49,50]. In MM, RANKL levels are increased, whereas OPG levels are decreased compared to normal individuals and patients with MGUS [39]. RANK is expressed on osteoclast precursor cells which are stimulated by RANKL [51]. RANKL is expressed by osteoblast and bone marrow stromal cells (55). OPG inhibits RANKL and also has a high affinity for RANKL [52]. Since osteoblasts express RANKL and secrete OPG, osteoblasts play an integral role in managing both bone formation and degradation. However, the higher is the ratio of RANKL to OPG, the worse is the prognosis [2]. Treatment with OPG or OPG-like molecules prevented both MM growth and bone destruction [38,53]. Recombinant OPG constructs, soluble RANK, OPG peptidomimetics [54,55], and, more recently, anti-RANKL antibodies such as denosumab have been developed to modulate the RANKL–OPG axis and reduce osteoclastic activity and myeloma [56,57,58].

Disruption of the BRC canopy impairs bone remodeling by allowing direct contact between the myeloma cells and the osteoclasts and osteoblasts [22]. Histological studies of iliac crest biopsies showed a direct correlation between the extents of the BRC canopy disruption with the magnitude of osteolytic lesions in patients with MM. A co-culture system with direct contact between myeloma cells and bone marrow stromal cells/pre-osteoblasts showed a significant decrease in OPG production, leading to the increased RANKL/OPG ratio, which results in increased bone degradation [39]. The direct contact between the stromal cells and the myeloma cells demonstrated increased secretion of IL-6 by the stromal cells [59]. IL-6 is known to activate osteoclast formation as well as increased myeloma cell proliferation [60]. Myeloma cells sometimes fuse with osteoclasts, forming myeloma–osteoclast hybrid cells that are more aggressive at eroding bone when compared to non-hybrid osteoclasts [61].

### 2.6. Decreased Osteoblastogenesis

In MBD, reduced bone formation secondary to decreased osteoblastic activity, leading to extensive bone loss and no repair, also plays a key role in the severity of the disease [62]. In early myeloma disease, the interaction of myeloma cells with the bone marrow microenvironment initiates the production of IL-1 and TNF-α. These cytokines recruit osteoblasts, leading to increased cell activity, which produces IL-6, a potent myeloma cell growth factor and bone resorption factor [63]. Osteoblasts also produce the growth factors IL-3 and GM–CSF, which further stimulate early myeloma cell growth and bone resorption. However, as the disease advances, BRC canopy is disrupted, leading to impaired synchrony between bone resorption and bone formation [22]. If the patient continues to have a high osteoblastic function, they do not develop MBD. Factors involved in the downregulation of osteoblastic activity mainly include Wnt/DKK1 pathway, secreted frizzle related protein–2 (SFRP-2), and Runx2. Other chemokines involved in decreasing osteoblastogenesis include hepatocyte growth factor (HGF), IL-7, sclerostin, and transforming growth factor-beta (TGF-β).

### 2.7. Wingless (Wnt) Signaling Pathway

The Wnt signaling pathway influences osteoblastogenesis and has significant involvement in bone formation and remodeling [64]. The Wnt signaling pathway is involved in embryogenesis, organ development after birth, and human tissue regeneration [65]. It also helps regulate stem cell production and CNS patterning. Studies have shown that the Wnt signaling pathway regulates cancer cell involvement and epidermal, intestinal, and hematopoietic systems [66,67]

Wnt genes encode Wnt family glycoproteins that transduce signals through frizzled (FZD) family receptors with extracellular Wnt-binding and cytoplasmic dishevelled-binding domains [68]. These Wnt glycoproteins are responsible for cell surface receptor activation, gene expression, cell proliferation, differentiation, and migration [69].

Wnts are classified as canonical if β-catenin dependent and noncanonical if β-catenin levels remain unaltered [70]. β-catenin is a major factor for OPG expression from osteoblasts [64]. Wnt signaling impacts osteoblastogenesis through a canonical pathway involving both intracellular and extracellular interactions, which is initiated by Wnt proteins binding to cell surface receptors made from a complex of lipoprotein related (LRP) 5/6 and FZD transmembrane proteins [71]. This complex induces an intracellular cascade involving dishevelled (DSH), Axin, and GSK-3, which prevents the forceful dilation of β-catenin, thus preventing its breakdown. Elevated β-catenin levels upregulate the transcription of genes involved in osteoblastic development. Of note, inactivation of the gene for LRP5 results in osteoporosis–pseudo-glioma syndrome, while gain-of-function mutation in LRP5 leads to a syndrome of hereditary high bone density [72,73]. These findings suggest that activation of this pathway causes increased osteoblastic activity and inhibition will decrease osteoblastogenesis [74]. Natural inhibitors of this pathway mainly include DKK1 and SFRP. Other regulators of the Wnt signaling pathway include Wnt inhibitory factor–1 (Wif-1), sclerostin, and sclerostin domain containing 1 (SOSTDC-1) [71].

### 2.8. Dickkopf-1 (DKK-1)

DKK1 is expressed by osteoblast and BMSC [75]. DKK1 has been shown to inhibit osteoblastogenesis via the Wnt pathway by inhibiting osteoblasts’ maturation and new bone formation. Studies have shown that DKK1 inhibits the Wnt signaling pathway by preventing the intracellular interaction, which protects β-catenin from opsonization and subsequent breakdown [76]. Mao et al. demonstrated that the mechanism of inhibition is through competitive binding to LRP6 and removal of trans-membrane protein receptors Kremens 1 and 2 [71,77]. This trimeric complex is then endocytosed, internalizing the receptor for Wnt proteins that are inhibiting the initiation of the Wnt signaling cascade. Ya-Wei Qiang et al. demonstrated that DKK1 inhibition of Wnt signaling indirectly stimulates osteoclastogenesis by inhibiting the maturation of osteoblasts which produce OPG (an inhibitor of osteoclastogenesis), resulting in decreased osteoclast inhibition [75]. In addition, they demonstrated that an increase in immature osteoblasts that produce RANKL stimulating osteoclast differentiation will no longer differentiate, causing a net increase in RANKL expression and subsequent osteoclast differentiation [78,79].

Studies such as those conducted by Tian et al. have shown that human plasma cells purified from bone marrow aspirates of myeloma patients expressed the gene for DKK1, and blood serum levels of DKK 1 were elevated in patients with MBD [80]. Expression of DKK 1 correlates with the stage of the disease, showing an increased level of DKK1 at more advanced stages. Other studies suggest that the DKK1 levels also correlate with the extent of lytic bone disease present [81].

### 2.9. Secreted Frizzled-Related Proteins (SFRP)

SFRP are other Wnt pathway antagonists that inhibit the binding of Wnt to the membrane-bound receptor, FZD, resulting in the downregulation of osteoblastic activity [62]. SFRP are a family of cysteine-rich glycoproteins which in combination with LRP 5/6 make up the cell membrane surface complex [82]. This complex inhibits Wnt signaling through interception and binding of Wnt proteins, preventing their interaction with the LRP 5/6 and FZD transmembrane proteins, which could initiate the canonical Wnt signaling cascade [71]. There are reports that they are expressed by several cells involved in bone formation and regulation, including myeloma cells and primary human osteoblasts [83,84].

SFRP-1 is consistently highly expressed by osteoblasts and suppress Wnt by 70% (91). SFRP-1 accumulates in pre-osteoblasts and declines upon maturation of the pre-osteoblast population. Thus, an increased number of osteoblastic precursors and reduced differentiation to mature osteoblasts lead to increased production of SFRP-1 [85]. This increased level of SFRP-1 reduces bone mineral density, trabecular volume, and biomechanical properties and increases osteochondral apoptosis [86]. Studies have shown that SFRP-2 is overexpressed specifically in myeloma cells derived from patients with advanced bone disease. Overexpression of SFRP-3 has been noticed with the progression of MGUS to myeloma [87]. Studies have shown that overexpression of SFRP-4 by osteoblast decreases the proliferation, resulting in decreased bone formation and viability [88].

### 2.10. Runt-Related Transcription Factor 2 (Runx2)/Core Binding Factor Runt Domain α Subunit 1 (CBFA1)

Runx2/CBFA1 is part of the non-canonical Wnt signaling pathway and constitutes a critical regulator of osteoblastogenesis, but this may also be affected by myeloma cells [89]. Runx2/CBFA1 plays an important role in the formation and differentiation of osteoblasts from mesenchymal cells and BMSC [23]. MM cells inhibit Runx2 activity in BMSC and osteoblast precursor cells, thereby impeding osteoblast differentiation [89]. Activation of Runx2/CBFA1 in human BMSC and preosteoblastic cells induce high expression of osteoblastic markers such as alkaline phosphatase and osteocalcin.

Co-cultures of human myeloma cells with mesenchymal cells showed an inhibitory effect on osteoblast formation and reduced expression of Runx2/CBFA1 by mesenchymal cells has been found after coming in direct contact with myeloma cells. In the same study, bone marrow biopsy specimens of myeloma patients with osteolytic lesions showed a markedly reduced number of Runx2/CBFA1 positive cells compared to those without myeloma bone disease [90,91].

Growth factor independence-1 (GFI-1), IL-7, and HGF are some factors that decrease Runx2/CBFA1 activity [92]. GFI-1 is a transcriptional depressor that binds to Runx2 and decreases its expression. IL-7 has a dual effect, increasing osteoclastic activity as well as inhibiting both early and late osteoblastic stimulation, differentiation, and maturation [89,93]. High levels of IL-7 have been demonstrated in the bone marrow of MM patients. IL-7 is also involved in the Runx2–mediated osteoblast suppression by inducing GFI-1 [92]. Overall, targeting Runx2, GFI-1, and IL-7 seems to have an encouraging result in overcoming MM-induced bone destruction. HGF is produced by myeloma cells and an elevated level of HGF has been demonstrated in the serum of MM patients compared to healthy individuals [94]. Besides, high levels of HGF are associated with poor prognosis. TGF-β released from the bone matrix during bone resorption inhibits osteoblastic differentiation and formation [59,95].

In recent studies, cysteine-rich 61 (CYR61/CCN1) protein, which is secreted in the bone marrow microenvironment, has been identified to stimulate osteoblastic differentiation by upregulating Runx2 in MM patients [96]. Studies have also shown that MM cells also overexpress Runx2, and higher levels of Runx2 in advanced stages of the disease are associated with poor prognosis [97]. Runx2 is also known to induce the AKT/β-catenin/survivin pathway along with the transcriptional activation of a gene panel that facilitates the homing of MM cells into the bone niche.

### 2.11. Extracellular Vesicles (EV) and Non-Coding RNA (ncRNA)

ncRNA includes ribonucleic acids that are not translated into proteins but participate in the translation process involving transfer RNAs and ribosomal RNAs, as well as splicing of small nuclear RNAs. These are considered the housekeeping ncRNAs. The other category includes inducible ncRNAs that are involved in the annealing process of complementary sequences in DNAs or RNAs and control gene expression. Various studies demonstrate that plasma cell neoplasms are regulated by several classes of ncRNAs [98,99]. The ncRNAs involved in the pathogenesis of MM bone disease are noted to be transported between cells by EVs [100]. Evidence has been building up in recent years that shows EVs and their ncRNAs are responsible for the onset of bone disease since they can promote osteoclast activation and inhibit osteogenesis by impacting the differentiation of mesenchymal stem cells [101,102,103,104,105].

## 3. Management of Multiple Myeloma Bone Disease

### 3.1. Radiotherapy

Malignant plasma cells exhibit increased sensitivity to radiation. For this reason, radiotherapy alone, without systemic chemotherapy, is considered the primary treatment modality for solitary osseous plasmacytomas. Radiotherapy has demonstrated excellent local control of osseous and extraosseous solitary plasmacytoma [106,107,108,109,110,111]. In MM patients, focal radiotherapy provides an effective modality of palliation for refractory bone pain, impending pathologic fractures, and to treat spinal cord compression. Active bone marrow containing areas, such as pelvic bones, should receive radiation in a judicious fashion if there is a need or plan for stem cell collection in the future.

### 3.2. Vertebroplasty or Kyphoplasty

These are minimal invasive procedures that include percutaneous injection of bone cement into fractured vertebral body for stabilization. Both these procedures are safe and effective in controlling pain and improving mobility in patients with compression fractures of vertebra from myeloma involvement [112,113].

## 4. Antiresorptive Therapies

### 4.1. Bisphosphonates

Bisphosphonates along with denosumab are the approved modalities of bone resorptive therapies for management of myeloma bone disease [114]. Commonly used bisphosphonates include zoledronic acid, pamidronate. and clodronate. Bisphosphonates, especially second-generation compounds which contain nitrogen moieties such as pamidronate or zoledronic acid, are several times more potent than non-nitrogen containing bisphosphonates (clodronate) [115]. These agents bind to hydroxyapatite and then cause osteoclast apoptosis by inhibiting mevalonate pathway via inhibition of farnesyl diphosphate (FPP) synthase, thereby disrupting prenylation of small intracellular guanine triphosphatases, which are essential for osteoclast function and survival [116,117,118].

In the United States, zoledronic acid is approved for treating myeloma bone disease at 4 mg intravenous dosing given every 3–4 weeks, while pamidronate is approved for 90 mg doses administered intravenously every 3–4 weeks. Both these agents have similar efficacy [119,120], and the selection is primarily based on administration time and additional benefits besides bone resorptive properties. Zoledronic acid is administered over 15 min while pamidronate is infused over 2 h. Zoledronic acid is not recommended for usage in severe renal impairment, whereas pamidronate can be used in patients with severe renal impairment by increasing infusion duration up to 4 h and using a reduced dose of 30 mg [121]. Zoledronic acid is preferred for management of patients with concomitant hypercalcemia. It is more effective in hypercalcemia reversal compared to pamidronate [122]. Uncommon but serious and important toxicities associated with bisphosphonate therapy include renal insufficiency and osteonecrosis of jaw [123]. Incidence of osteonecrosis of jaw is higher with zoledronic acid compared to pamidronate (10% vs. 4%) [124]. Major risk factors for osteonecrosis of jaw on bisphosphonate treatment include poor oral hygiene, invasive dental procedures, and local infections [125,126]. Other non-serious toxicities include flu-like symptoms due to an acute phase reaction.

Bisphosphonates have been employed in the management of MM-related bone disease since the early 1980s, with increased acceptance and usage over the decades, to being considered now as an essential component of MM management. Intravenous pamidronate at 90 mg every month demonstrated efficacy compared to placebo, and it was also confirmed to be effective in relapsed or refractory patients [116,127]. A study published in 2010 showed that pamidronate at 30 mg/month was as effective as a 90 mg/month dose in reducing time to skeletal-related events and skeletal-related event-free survival [128]. Zoledronic acid established superiority over clodronate in a large randomized controlled trial (MRC Myeloma IX) [129,130]. This trial and later the secondary analysis established the superiority of zoledronic acid over clodronate in all patient subsets including transplant or nontransplant candidates, patients receiving thalidomide maintenance or not, and most importantly in patients presenting with or without bone lesions. The secondary analysis highlights the importance of adding bisphosphonates upfront along with myeloma therapy in all newly diagnosed patients. The results from MRC Myeloma IX trial show a significant increase in progression-free survival and overall survival for patients receiving zoledronic acid compared to clodronate, regardless of whether they had bone lesions. This resulted in ASCO putting forward guidelines recommending initiation of bisphosphonate therapy in any newly diagnosed myeloma patient who is on active myeloma therapy [131].

Studies have been conducted to assess the optimal dosing interval of zoledronic acid, including extending from monthly to every 3 months dosing while maintaining efficacy. The Z-MARK study evaluated efficacy and safety of every 12 weeks dosing of zoledronic acid compared to every 4 weeks dosing in patients who had received 1–2 years of prior monthly bisphosphonate therapy [132]. The overall incidence of skeletal-related events in the second year was low at 4.9% and the 2-year incidence of osteonecrosis of jaw was 3.3%. Another large randomized controlled trial comparing every 12 weeks to a 4-week regimen of zoledronic acid in 1544 patients with MM or bone metastasis from solid malignancies analyzed 278 patients with MM [133]. There was no difference in skeletal-related events, osteonecrosis of jaw, or renal dysfunction among patients in either treatment groups. However, a high study dropout rate was noted in this study. Both these studies suggest a possible feasibility of less frequent zoledronic acid administration, and the final decision is still based on physician discretion and patient preference.

According to the International Myeloma Working Group (IMWG), bisphosphonates can be given until disease progression in patients who do not achieve a complete response (CR) or very good partial response (VGPR) [134]. For those patients with CR or VGPR, IMWG currently recommends 12–24 months of bisphosphonate treatment and further continuation based on physician discretion. The MRC Myeloma IX trial reported a small number of patients who received bisphosphonate therapy for up to 5 years, who showed continued benefit in preventing incidence of skeletal-related events [129,130]. However, incidence of osteonecrosis of jaw also continued to increase in these patients over this time period. In MM patients with at least VGPR, the optimal duration of bisphosphonates is an ongoing area of active research.

According to a meta-analysis conducted by Cochrane database, bisphosphonates have to be administered to 6–15 myeloma patients to prevent one skeletal-related event [135].

### 4.2. Denosumab

Denosumab is a fully humanized monoclonal antibody that targets RANK-ligand (RANKL), which was approved in 2018 by the FDA for treatment of myeloma bone disease. The RANK–RANKL system is an important pathway in regulation of normal as well as pathologic bone remodeling [136]. RANK–RANKL interaction mediates osteoclast precursors, thereby promoting differentiation into osteoclasts as well as activating mature osteoclasts to resorb bone. Denosumab binds to RANKL with high affinity, thereby preventing activation of RANK and thus inhibiting formation, activation, and survival of osteoclasts, which results in reduction of bone resorption as well as bone destruction in MM.

A randomized trial conducted by Henry et al., comparing denosumab with zoledronic acid in patients with bone metastasis from advanced cancers (excluding breast and prostate cancer) or MM, included 180 patients with MM [56]. Subgroup analysis of this population demonstrated favorable survival with zoledronic acid. In another larger randomized controlled trial aimed at comparing denosumab to zoledronic acid in patients with MM, denosumab was found to be non-inferior to zoledronic acid in delaying skeletal-related events [137]. Overall survival was found to be similar in both arms, but median progression-free survival was 10.7 months longer for patients receiving denosumab. The denosumab arm was associated with higher incidence of hypocalcemia (17% vs. 12%), lower rates of renal dysfunction, and higher rates of doubling of creatinine from baseline (3% vs. 7%), compared to zoledronic acid. Rates of osteonecrosis of jaw were low in both arms and not statistically different (3% vs. 2%).

For patients receiving denosumab or bisphosphonates, regular monitoring of kidney function and dental hygiene along with vitamin D and calcium supplementation is recommended.

## 5. Systemic Anti-Myeloma Treatments

### 5.1. Proteasome Inhibitors

Proteasome pathway inhibition has been shown to be involved in regulation of bone remodeling. Proteasome-dependent inhibition of NF-ƙB leads to a reduction in RANKL-mediated osteoclast differentiation [138,139]. The proteasome pathway also plays an important role in osteoblast differentiation and inhibiting it induces new bone formation. In MM mouse models, proteasome inhibitors induced new bone formation by increasing expression of BMP2, a potent osteoblast differentiation inducing agent [140,141,142].

Bortezomib is a highly effective proteasome inhibitor which is currently the mainstay of anti-myeloma treatment regimens [143,144,145,146,147]. Several preclinical [148,149,150,151,152] and clinical studies [153,154,155,156,157,158] have investigated the effect of bortezomib on bone remodeling. Based on evidence from preclinical studies, clinical effects of bortezomib on bone remodeling could be analyzed by direct measurements of bone characteristics. For example, bortezomib-based treatment was shown to increase serum alkaline phosphatase (ALP), bone ALP, and serum parathyroid hormone (PTH) levels, as well as reduce levels of DKK-1 (marker of bone resorption). Some of these clinical studies have also indicated that changes in these bone remodeling markers were noted in both responders and non-responders to bortezomib-based therapy. In a randomized phase III trial (VISTA) that compared bortezomib, melphalan, and prednisone (VMP) regimen to melphalan and prednisone (MP), the rates of bisphosphonate usage, progression of bone disease, and needs for palliative radiation therapy were all lower in the VMP arm compared to the MP arm [154]. In another study evaluating bortezomib, thalidomide, and dexamethasone (VTD) regimen as consolidation in patients with newly diagnosed MM, it was demonstrated that bortezomib-based therapy was associated with RANKL/OPG ratio normalization and achievement of a very low incidence rate of skeletal-related events (2%), even without concomitant use of bisphosphonates [156].

Carfilzomib is a second-generation proteasome inhibitor, which is currently FDA approved for use in relapsed/refractory MM as part of triplet regimen or doublet regimen [159,160,161,162,163,164]. It is also used off-label in newly diagnosed MM patients (in both transplant and non-transplant candidates) and is included in the National Comprehensive Cancer Network (NCCN) guidelines for the same indications [165,166,167,168]. In pre-clinical studies, carfilzomib demonstrated activity against bone resorption and promoted new bone formation [142,169,170]. It was also observed that carfilzomib was more effective in enhancing osteoblastic activity compared to bortezomib, suggesting that carfilzomib may be a more potent promoter of new bone formation. A small phase II study by Suvannasankha et al. explored the effect of single agent carfilzomib on bone metabolism in patients with relapsed MM [171]. Only 10 patients were enrolled in this study, which demonstrated bone anabolic effects along with inhibition of bone resorption. A retrospective analysis of two phase II trials using carfilzomib as a single agent in relapsed/refractory myeloma (PX-171-003 and PX-171-004) indicated that an early elevation in alkaline phosphatase is associated with subsequent myeloma response [172].

### 5.2. Immunomodulatory Drugs (IMiDs)

IMiDs form an important group of drugs in MM therapy arsenal. They are thalidomide analogs, which induce apoptosis of myeloma cells and enhance antimyeloma T-cell and NK-cell immunity [173,174]. IMiDs also inhibit angiogenesis, thereby making the bone marrow microenvironment inconducive for myeloma cell growth and survival [175]. Bone directed effects of IMiDs have been studied with conflicting results. In a pre-clinical study, pomalidomide (previously known as CC-4047) inhibited osteoclastogenesis through downregulation of the hematopoietic transcriptional factor PU.1 [176]. Another in vitro study demonstrated inhibition of osteoblastic activity by IMiDs, as shown by reduced mineralization and alkaline phosphatase activity [177]. In a clinical setting, treatment of MM with thalidomide and dexamethasone in relapsed/refractory setting resulted in normalization of RANKL/OPG ratio [178]. Similarly, lenalidomide also demonstrated reduced osteoclastic bone resorption by inhibiting osteoclast-activating factors APRIL (a proliferation inducing ligand) and BAFF (B-cell activating factor) [179].

## 6. Novel Approaches and Future Directions

### 6.1. Bruton Tyrosine Kinase Inhibitors (BTKi)

Bruton tyrosine kinase (BTK) is a non-receptor tyrosine kinase, which is expressed in many hematopoietic cells, including maturing B-cells, and plays an important role in B-cell maturation and function [180,181]. BTK inhibition has proven to be a highly successful therapeutic target in management of various B-cell malignancies such as chronic lymphocytic leukemia and mantle cell lymphoma [182,183]. BTK has been shown to be strongly expressed in myeloma cells and selectively expressed in osteoclasts but not in osteoblasts [184,185]. Based on this physiologic principle, investigators demonstrated that BTKi such as ibrutinib reduced MM tumor burden while limiting osteoclastogenesis and bone resorption. This principle needs to be tested further in clinical studies.

### 6.2. Anti-DKK1

DKK1 is expressed by osteoblasts, and it acts as an antagonist of Wnt pathway, which results in inhibition of osteoblast maturation and new bone formation [75]. High expression of DKK1 is noted in patients with MM and extensive bone involvement. In a pre-clinical study using SCID-rab mice, it was demonstrated that anti-DKK1 decreased osteoclastogenesis and promoted new bone formation by stimulating osteoblast activity, in both myeloma-involved and uninvolved bones [186].

### 6.3. OPG Agonists

Osteoprotogerin (OPG) is secreted by osteoblasts, bone marrow stromal cells, and endothelial cells. OPG blocks the interaction between RANK and RANKL, thereby blocking activation and differentiation of osteoclasts [51,187]. OPG agonists mimic this activity by acting as a decoy receptor for RANKL. Safety and efficacy of OPG agonists (AMGN 0007) was evaluated in a phase I study. In this study, patients with MM and breast cancer patients with bone lesions were treated with a single dose of AMGN 0007 or pamidronate at 90 mg. The efficacy was assessed by measuring levels of bone resorption marker NTX. AMGN 0007 results in decreased levels of NTX comparable to pamidronate, and it was tolerated well [55].

### 6.4. Anti-Sclerostin

Sclerostin is a cysteine knot-containing protein, produced by osteocytes. It induces apoptosis of mature osteoblasts by activating the caspase pathway and inhibits osteoblast-driven bone formation [188]. Romosozumab, a humanized anti-sclerostin antibody, was found to be effective in management of benign bone disorders [189]. This rationale is further being tested in MM models, currently in preclinical stages, especially in combination with antineoplastic agents such as proteasome inhibitors [190,191].

### 6.5. TGF-β

TGF-β has been implicated in tumor-induced bone disease in various cancers [192]. The exact mechanism of TGF-β-induced bone disease is unknown. In a preclinical model, a TGF-β inhibitor, SRI31277, was administered to mice with multiple osteolytic lesions and was shown to decrease the tumor burden and decrease phosphorylated SMAD2, which was associated with decrease in osteoclasts and increase in osteoblasts [193]. This would be a useful approach in myeloma bone disease management, as well as skeletal disease management in various other cancers, if proven to be effective in humans.

### 6.6. Activin A and Sotatercept

Activin A is a member of the TGF-β superfamily, which is released from osteoblast and osteoclast precursors and has been shown to be elevated in patients with myeloma bone disease. Activin A antagonizes bone morphogenetic proteins (BMPs) by competing for their receptors and therefore inhibits BMP-induced apoptosis of malignant plasma cells [194,195]. Sotatercept, a soluble recombinant activin receptor type IIA (ActRIIA) ligand fused to human Fc-Ig fragment, binds activin A/B as well as members of the TGF-β superfamily and disrupts downstream cascades. In a phase I trial, Yee et al. demonstrated that sotatercept in combination with lenalidomide and dexamethasone was well tolerated; preliminary data suggest that sotatercept leads to early increases in both hemoglobin and bone mineral density, and it was noted to be the first agent that may address both these significant morbidity issues in MM [196]. In a phase II trial, sotatercept in addition to melphalan, prednisolone, and thalidomide resulted in an increase in bone alkaline phosphatase, indicating improved bone turnover [195].

### 6.7. Radionuclides

Radionuclides or radiopharmaceuticals demonstrate affinity for bone undergoing active remodeling and therefore can deliver localized therapeutic effect. This has been studied well in the management of metastatic prostate cancers with bone disease, using radium-223 and samarium-153 [197,198]. Samarium-153 has been evaluated in patients with myeloma bone disease and bone pain, with significant improvement in bone pain [199].

### 6.8. Recombinant Parathyroid Hormone (rPTH)

The role of rPTH, a teriparatide, in increasing bone mineral density is controversial because of conflicting evidence that rPTH can also stimulate osteoclastogenesis [200]. In addition, there have been reports of malignancies occurring in patients on rPTH, including emergence of myeloma in a patient with osteoporosis treated with rPTH. These concerns have for now halted further extrapolation of rPTH in the management of MBD [201,202].

## 7. Conclusions

Over the years, and most notably within the last decade, treatment of MM has become more effective with the incorporation of various novel therapies such as proteasome inhibitors, immunomodulatory drugs, monoclonal antibodies, histone deacetylase inhibitors, selective inhibitors of nuclear export, and the latest anti-BCMA therapy. Therefore, it is imperative to develop better supportive strategies, including more effective management of myeloma bone disease, to match the effectiveness of newer and more effective anti-myeloma therapies. Bisphosphonates and more recently denosumab have been the mainstay of current MBD management. With a better understanding of the complex biology of myeloma bone disease, development of treatments aimed at targeting the bone and marrow microenvironment will be able to treat myeloma effectively while preserving bone health and potentially improving overall disease outcomes.
